# The 4q27 locus and prostate cancer risk

**DOI:** 10.1186/1471-2407-10-69

**Published:** 2010-02-25

**Authors:** Elizabeth A Tindall, Hoa N Hoang, Melissa C Southey, Dallas R English, John L Hopper, Graham G Giles, Gianluca Severi, Vanessa M Hayes

**Affiliations:** 1 Cancer Genetics Group, Children's Cancer Institute Australia for Medical Research, Sydney Children's Hospital, High St, Randwick, NSW, Australia; 2 Faculty of Medicine, University of New South Wales, Randwick, NSW Australia; 3 Cancer Epidemiology Centre, The Cancer Council Victoria, Carlton, Vic, Australia; 4 Department of Pathology, University of Melbourne, Melbourne, Vic, Australia; 5 Centre for Molecular, Environmental, Genetic, and Analytical Epidemiology, University of Melbourne, Melbourne, Vic, Australia

## Abstract

**Background:**

Chronic inflammation is considered to be implicated in the development of prostate cancer. In this study we are the first to investigate a potential association between variants in an autoimmune related region on chromosome 4q27 and prostate cancer risk. This region harbors two cytokine genes *IL-2 *and the recently described *IL-21*.

**Methods:**

We genotyped six variants previously associated with autoimmune disease (namely rs13151961, rs13119723, rs17388568, rs3136534, rs6822844 and rs6840978) and one functional *IL-2 *promoter variant (rs2069762) for possible association with prostate cancer risk using the Australian Risk Factors for Prostate Cancer case-control Study.

**Results:**

Overall, our results do not support an association between the seven variants at position 4q27 and prostate cancer risk. Per allele odds ratios (ORs) were not significantly different from 1 (all P-values = 0.06). However, we found suggestive evidence for a significant association between the presence of the rs13119723 variant (located in a protein of unknown function) and men with a family history of prostate cancer in first-degree relatives (P-value for interaction 0.02). The per allele OR associated with this variant was significantly higher than 1 (2.37; 95% C.I. = 1.01-5.57).

**Conclusions:**

We suggest that genetic variation within the chromosome 4q27 locus might be associated with prostate cancer susceptibility in men with a family history of the disease. Furthermore, our study alludes to a potential role of unknown protein KIAA1109 in conferring this risk.

## Background

Prostate cancer is the most common cancer effecting men in Western society and is the second highest contributor to cancer-related deaths world-wide [1]. Despite such vast prevalence little is known about the etiology of prostate cancer. To date, there are three well-defined risk factors, which include increased age, ethnicity and a family history of the disease [2]; evidence for the latter supporting a genetic contribution to prostate cancer risk. More recently, inflammation has been implicated in prostate cancer pathogenesis [3].

Different forms of prostate specific inflammation, including chronic prostatitis and proliferative inflammatory atrophy (PIA) have been frequently associated with the development of prostate cancer [4,5]. As an understanding of the role of inflammation in the etiology of prostate cancer begins to emerge, an imbalance in cytokine production is thought to contribute to chronic inflammation and a failed tumor immune response. Suggestions that these specific inflammatory mediated conditions may in fact be pre-cursor lesions to prostate cancer development have prompted numerous investigations into identifying inflammatory gene variants that might predispose individuals to prostate cancer risk. (reviewed in [6]).

**Figure 1:**
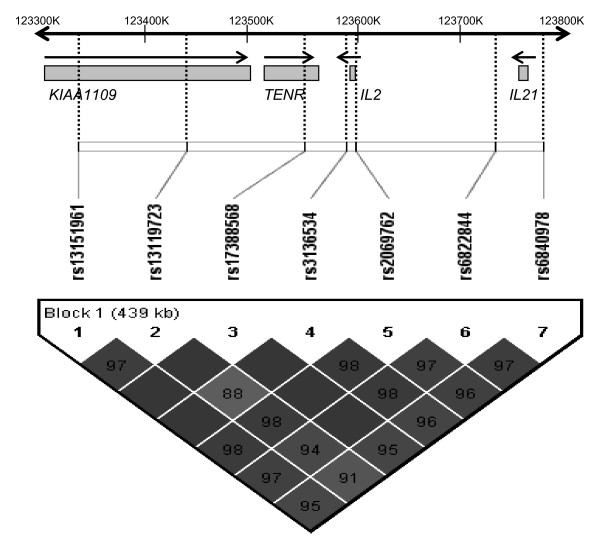
**Pairwise LD for gene variants located within approximately 480 kb at chromosomal position 4q27**. The 4q27 region harbors cytokine coding genes *IL-2 *and *IL-21*, testis nuclear RNA-binding protein coding gene *TENR *and a gene coding for a protein of unknown function, *KIAA1109*. The seven selected SNPs (indicated by rs numbers) in this study display tight linkage across the region. The reference genome assembly used to determine physical SNP position was NCBI Build version 36.3.

A 480 kilo base (kb) block of linkage disequilibrium (LD) at chromosomal position 4q27 (Figure 1), harbors two cytokine genes, *Interleukin *(*IL*)*-2 *and *IL-21*. These genes encode proteins involved in numerous immune functions such as the activation and proliferation of immune cells including B-, T- and natural killer (NK) cells. IL-2, which is produced primarily by activated CD4+ effector T-cells, is a member of the common γ-chain cytokine family and appears to have pleiotropic effects in the immune system, acting as both a pro- and anti-inflammatory regulator [7]. Similarly, IL-2 has been described as contributing to both pro- and anti-tumor immunity. In models of melanoma and renal cell carcinoma, administration of a high dose IL-2 based immunotherapy correlates with an elevated T regulatory (Treg) cell response associated with down regulation of tumor specific immunity [8]. Alternatively lower doses of IL-2 for treatment of common malignancies including prostate cancer contributes to an anti-tumor immune response via activation of tumor specific NK cells [9-11]. A balanced IL-2 cytokine production in the prostate microenvironment may thus contribute to prostate cancer susceptibility. To date, a single prostate cancer association study has investigated genetic variants within *IL-2 *for prostate cancer risk and revealed a significant contribution of a synonymous *IL-2 *exon 1 variant (rs2069763) to disease susceptibility [12].

IL-21 is the most recently described member of the common γ-chain cytokine family. Similarly to IL-2, IL-21 is expressed by activated CD4+ T-cells and has been described as having anti-tumor effects in mouse models for several different malignancies, including prostate cancer [13]. Experimental models have demonstrated the anti-tumor potential of IL-21, via expansion of tumor specific CD8+ effector T cells and suppression of anti-inflammatory Treg cells [14]. IL-21 is involved in T-helper type 17 (Th17) effector cell function [15], which has been implicated in prostate cancer development [16] and more recently with progression to metastasis [17]. No studies have to date investigated the potential role of *IL21 *variation in prostate cancer susceptibility.

In addition to *IL-2 *and *IL-21*, the 480 kb region at 4q27 encompasses genes encoding testis nuclear RNA-binding protein (*TENR*) and a protein of unknown function, *KIAA1109*. The chromosomal region at position 4q27 has been highlighted as a potential risk locus in genome-wide association studies (GWAS) and candidate gene studies for several common inflammatory disorders including celiac disease, type 1 diabetes, rheumatoid arthritis, systemic lupus erythematosus (SLE), psoriasis, multiple sclerosis and both forms of inflammatory bowel disease (IBD), ulcerative colitis and Chron's disease [18-24]. Although replication studies have confirmed 4q27 as a risk locus for inflammatory disorders [22,24-29], tight linkage disequilibrium (LD), essentially responsible for identification of the 4q27 region as a prostate cancer risk locus, in turn limits identification of the true casual variant.

The proposed link between the 4q27 locus and autoimmune disease susceptibility is suggestive of a functional role for this region in maintaining a balanced immune response. Under the proposed hypothesis that chronic inflammation may mediate prostate cancer development, further supported by casual observations of tentative association between the incidence of prostate cancer and several common immune disorders including ulcerative colitis [30], rheumatoid arthritis [31], Crohn disease [32] and asthma [33], we investigate a potential role for the 4q27 region in prostate cancer susceptibility. Six autoimmune associated variants and a single functional variant were genotyped using a large Australian-based case-control study of prostate cancer.

## Methods

### Study Population

The Risk Factors for Prostate Cancer (RFPC) study is a population-based case-control study of 825 cases and 734 controls from the Melbourne and Perth metropolitan areas of Australia, and defined in Additional file 1. The men of predominantly European descent (including 9 cases and 13 controls of Asian origin) were recruited between 1994 and 1998 and described in detail elsewhere [34]. Eligible cases were diagnosed with histopathologically confirmed prostate cancer via the State Cancer Registries and included irrespective of familial status. Cases were diagnosed before age 70 years with a Gleason Score >4. Tumor stage (stage I to IV) and grade (moderate: Gleason 5-7; high: Gleason 8-10) were obtained from histopathological reports. Controls were randomly selected from the State Electoral Rolls and frequency matched to the expected age distribution of cases. Information including age, family history of prostate cancer (in first-degree relatives), country of birth, and lifestyle were recorded during a face-to-face interview. Ethics approval for this study was obtained from The Cancer Council Victoria Human Research Ethics Committee, Australia (HREC0808).

### Variant Selection and Genotyping

Seven single nucleotide variants across the 4q27 locus were selected for analysis with prostate cancer risk. Six (rs13151961, rs13119723, rs17388568, rs3136534, rs6822844 and rs6840978) were chosen due to highly significant associations reported in previous studies for autoimmune disease susceptibility. These associations may reflect an as yet unknown biological effect of these variants. The *IL-2 *promoter variant (rs2069762) was specifically selected based on previous functional reports [35,36].

The six variants rs13151961, rs13119723, rs17388568, rs3136534, rs6822844 and rs6840978 were genotyped using Applied Biosystem 7900HT TaqMan allelic discrimination method, with an average 11.5% random study replication. The *IL-2 *promoter variant rs2069762, was genotyped on two platforms using the Sequenom MassArray using homogenous MassExtend (hME) and TaqMan allelic discrimination methods. Study replication was performed for the purpose of platform and chemistry assessment and quantification, as previously published [37].

### Statistical Analysis

Allele frequency estimates and tests of deviation from Hardy-Weinberg equilibrium (HWE) were carried out using standard procedures based on asymptotic likelihood theory [38]. Unconditional logistic regression analysis was used to test for independence between the variants and age (<55, 55-59, >60), country of birth (Australia, others) and family history of prostate cancer (affected first-degree relative and two or more first degree relatives affected). Case-control analyses were conducted using unconditional logistic regression and OR estimates and their 95% confidence intervals (CI) were derived under likelihood theory. We fitted dominant, recessive, co-dominant, and per allele models. Heterogeneity in the odds ratios by age and family history of prostate cancer in first-degree relatives (0 or at least one) was tested by including an interaction term in the logistic model. One polytomous logistic regression model was used to estimate odds ratios (OR) for stage I-II and stage III-IV tumors and another to estimate ORs for moderate-grade and high-grade tumors. In order to test for heterogeneity in the ORs by tumor stage or grade, the likelihood from these models was compared with that from polytomous logistic regression models with ORs constrained not to vary by tumour stage or grade. All statistical analyses were performed using Stata 10 (Stata Corporation). All tests were two-sided and a 5% level was used as the threshold for statistical significance.

## Results

Genotype calls were achieved for more than 99% of the study samples, with a 100% genotyping replication concordance using TaqMan allelic discrimination. Genotype distributions were consistent with HWE for cases and controls as well as cases and controls combined (all P > 0.19). No significant association was observed between genotypes and tumor stage or grade, age of prostate cancer diagnosis, prostate-specific antigen (PSA) levels, or country of birth for any of the variants we assessed in this study.

Overall we found no significant association between the seven variants and prostate cancer risk (Additional file 2). The per-allele ORs ranged from 0.92 (95% C.I. = 0.79-1.07) to 1.16 (95% C.I. = 1.00-1.35) and they were all not significantly different from 1 (P = 0.06). The ORs were virtually unchanged after excluding the 22 men that were born in Asia. For rs3136534, the dominant model showed marginal evidence of association with risk: the OR for carriers of the rare allele versus carriers of two copies of the common allele was 1.23 (95% C.I. = 1.00-1.50, P-value = 0.046). Analysis by tumor stage, grade and age did not show any significant association. Analysis by family history of prostate cancer (Additional file 3) however showed evidence that the G allele for the intronic *KIAA1109 *variant rs13119723, was significantly more frequent in men with a family history compared to those with no family history (P-value for interaction = 0.02). The per allele OR associated with this variant was significantly higher than 1 (2.37; 95% C.I. = 1.01-5.57) for men with a family history of prostate cancer.

Previous studies have reported a strong degree of LD within the 4q27 gene locus. Our results similarly support a high degree of linkage within this region (Figure 1).

## Discussion

Results using the RFPCS suggest that genetic variation at chromosomal region 4q27 is not associated with overall prostate cancer risk. At 0.05 level of significance under a dominant model the sample size of our study allows to detect with 80% power a minimum OR of 1.33 for the most common SNP (rs2069762) and 1.36 for the least common SNP (rs13151961). The corresponding minimum detectable ORs under a recessive model are 1.54 and 2.11. Analysis of men with a family history of the disease, however, indicates that this region may be a risk locus for familial prostate cancer.

The potential role of chronic inflammation in prostate cancer development, together with an observed association between the 4q27 region and susceptibility to autoimmune disease and in turn immune dysfunction, implicates the cytokine rich 4q27 region as a candidate prostate cancer risk locus. IL-2 and IL-21 are involved in regulating an immune response at multiple intervals, thus genetic variation likely to influence the function of these cytokines may have catastrophic effects to host immunity.

Although *IL-2 *is well studied for association with autoimmune diseases, only a single study has investigated genetic association with risk for prostate cancer, implicating an indirect prostate cancer susceptibility marker [12], with no direct functional significance [39]. In our study we elected to screen the promoter variant rs2069762, shown to influence IL-2 production up to 3-fold [35,36] for a possible direct association. Although our results show no significant association for rs2069762, this is the first study to address the potential role of this variant in prostate cancer risk. The variant rs3136534 in the *IL2 *3' untranslated region (UTR) however, displayed a marginally significant association with risk for prostate cancer.

The *IL-21 *gene is still considered a relatively newly discovered cytokine, and thus few disease association studies have been reported. Candidate gene studies have identified single variants (not observed on GWAS) within *IL-21 *to be associated with autoimmune diseases including SLE [40], type 1 diabetes [41] and atopic asthma [42]. The latter study identified a synonymous exon 3 variant (rs4833837), also reported to correlate with serum levels of immunoglobulin (Ig)E and IL-21 [42]. These results further implicate IL-21 in maintaining and regulating an immune response, although a high degree of LD within this gene region has hindered efforts to narrow the locus to its true causal variants. Despite suggestions that rs4833837 is associated with differential IL-21 activity, as yet no direct functional role for any *IL-21 *variant has been reported. In this study we selected variants that were most significantly associated with autoimmune disease susceptibility and are the first, to our knowledge to investigate variants within the *IL-21 *gene region for prostate cancer risk.

A family history of prostate cancer has long been accepted as a major contributor to an increased risk of developing the disease [2]. Identification of variants responsible for this inherent risk can be achieved by assessing variant frequency in cases with affected relatives. For the *KIAA1109 *rs13119723 variant, we found an increased OR for men with a family history of prostate cancer. This result reflects a different genotype distribution for both cases and controls with a family history compared with cases and controls with no family history. However, we cannot exclude the possibility that the increased OR may be due to chance and to the relatively small number of men with a family history of prostate cancer in our study.

Although prostate cancer GWAS have thus far failed to implicate the 4q27 region as a candidate risk locus, the growing number of candidate gene regions already identified in these large-scale studies, highlights the complex and heterogeneous nature of prostate cancer. Thus, multiple, low to moderate risk alleles (not yet identified by GWAS) may still be implemented in the disease. It could therefore be argued that our pathway-linked gene approach to identify modest risk loci, in combination with large scale GWAS, may be appropriate to ensure complete coverage of potential prostate cancer risk factors.

## Conclusions

To establish whether the 4q27 cytokine rich region is a locus for familial prostate cancer risk as suggested in this study, the association needs to be replicated in independent studies that include a larger number of men with a family history of the disease. In addition to harboring a gene of as yet unknown function, few functionally relevant variants within this region have been elucidated. Hence, a more comprehensive analysis of potentially functional variants within 4q27 may provide a more accurate approach for analysis of prostate cancer susceptibility.

## Competing interests

The authors declare that they have no competing interests.

## Authors' contributions

EAT participated in the design of the study, performed all genotyping and drafted the manuscript. The data contained within will contribute partial fulfillment of the requirements for the Degree of Doctor of Philosophy for EAT. MCS, DRE, JLH and GGG were responsible for collection and maintaining the epidemiological resource. HNH and GS performed statistical analysis and helped draft the manuscript. VMH facilitated the study design, analysis and drafted manuscript. All authors read and approved the final manuscript.

## Pre-publication history

The pre-publication history for this paper can be accessed here:

http://www.biomedcentral.com/1471-2407/10/69/prepub

## Supplementary Material

Additional file 1 **Table S1**. Characteristics of participants in the Australian Risk Factors for Prostate Cancer Study Click here for file

Additional file 2 **Table S2**. Allele and genotype distributions for seven variants across chromosome 4q27 and prostate cancer risk Click here for file

Additional file 3 **Table S3**. Association between variants and prostate cancer risk by family history of prostate cancer Click here for file

## References

[B1] Baade PD Youlden DR Krnjacki LJ International epidemiology of prostate cancer: geographical distribution and secular trends Mol Nutr Food Res 2009 53 2 171 184 10.1002/mnfr.200700511 19101947

[B2] Fleshner NE Lawrentschuk N Risk of developing prostate cancer in the future: overview of prognostic biomarkers Urology 2009 73 5 Suppl S21 27 10.1016/j.urology.2009.02.022 19375623

[B3] Vasto S Carruba G Candore G Italiano E Di Bona D Caruso C Inflammation and prostate cancer Future Oncol 2008 4 5 637 645 10.2217/14796694.4.5.637 18922121

[B4] De Marzo AM Platz EA Sutcliffe S Xu J Grönberg H Drake CG Nakai Y Isaacs WB Nelson WG Inflammation in prostate carcinogenesis Nature Reviews Cancer 2007 7 256 269 10.1038/nrc2090 17384581 PMC3552388

[B5] Sandhu JS Prostate cancer and chronic prostatitis Curr Urol Rep 2008 9 4 328 332 10.1007/s11934-008-0056-6 18765133

[B6] Sun J Turner A Xu J Grönberg H Isaacs W Genetic variability in inflammation pathways and prostate cancer risk Urological Oncology 2007 25 3 250 259 10.1016/j.urolonc.2006.10.001 17483024

[B7] Hoyer KK Dooms H Barron L Abbas AK Interleukin-2 in the development and control of inflammatory disease Immunol Rev 2008 226 19 28 10.1111/j.1600-065X.2008.00697.x 19161413

[B8] Vliet HJ van der Koon HB Yue SC Uzunparmak B Seery V Gavin MA Rudensky AY Atkins MB Balk SP Exley MA Effects of the administration of high-dose interleukin-2 on immunoregulatory cell subsets in patients with advanced melanoma and renal cell cancer Clin Cancer Res 2007 13 7 2100 2108 10.1158/1078-0432.CCR-06-1662 17404092

[B9] Brill TH Kubler HR Pohla H Buchner A Fend F Schuster T von Randenborgh H Paul R Kummer T Plank C Therapeutic Vaccination with an IL-2-IFNgamma-secreting Allogeneic Tumor Vaccine in Patients with Progressive Castration-Resistant Prostate Cancer - a Phase I/II Trial Hum Gene Ther 2009 19671000 10.1089/hum.2009.101

[B10] Hallett WH Ames E Alvarez M Barao I Taylor PA Blazar BR Murphy WJ Combination therapy using IL-2 and anti-CD25 results in augmented natural killer cell-mediated antitumor responses Biol Blood Marrow Transplant 2008 14 10 1088 1099 10.1016/j.bbmt.2008.08.001 18804038 PMC2735407

[B11] Lechleider RJ Arlen PM Tsang KY Steinberg SM Yokokawa J Cereda V Camphausen K Schlom J Dahut WL Gulley JL Safety and immunologic response of a viral vaccine to prostate-specific antigen in combination with radiation therapy when metronomic-dose interleukin 2 is used as an adjuvant Clin Cancer Res 2008 14 16 5284 5291 10.1158/1078-0432.CCR-07-5162 18698048 PMC2639763

[B12] Wu H-C Chang C-H Wan L Wu C-I Tsai F-J Chen W-C IL-2 gene C/T polymorphism is associated with prostate cancer Journal of Clinical Laboratory Analysis 2006 20 6 245 249 10.1002/jcla.20149 17115417 PMC6807315

[B13] Takaki R Hayakawa Y Nelson A Sivakumar PV Hughes S Smyth MJ Lanier LL IL-21 enhances tumor rejection through a NKG2D-dependent mechanism J Immunol 2005 175 4 2167 2173 16081783 10.4049/jimmunol.175.4.2167

[B14] Kim-Schulze S Kim HS Fan Q Kim DW Kaufman HL Local IL-21 promotes the therapeutic activity of effector T cells by decreasing regulatory T cells within the tumor microenvironment Mol Ther 2009 17 2 380 388 10.1038/mt.2008.249 19034262 PMC2835064

[B15] Huber M Brustle A Reinhard K Guralnik A Walter G Mahiny A von Low E Lohoff M IRF4 is essential for IL-21-mediated induction, amplification, and stabilization of the Th17 phenotype Proc Natl Acad Sci USA 2008 105 52 20846 20851 10.1073/pnas.0809077106 19088203 PMC2634912

[B16] Sfanos KS Bruno TC Maris CH Xu L Thoburn CJ DeMarzo AM Meeker AK Isaacs WB Drake CG Phenotypic analysis of prostate-infiltrating lymphocytes reveals TH17 and Treg skewing Clin Cancer Res 2008 14 11 3254 3261 10.1158/1078-0432.CCR-07-5164 18519750 PMC3082357

[B17] Derhovanessian E Adams V Hahnel K Groeger A Pandha H Ward S Pawelec G Pretreatment frequency of circulating IL-17+ CD4+ T-cells, but not Tregs, correlates with clinical response to whole-cell vaccination in prostate cancer patients Int J Cancer 2009 125 6 1372 1379 10.1002/ijc.24497 19533748

[B18] Genome-wide association study of 14,000 cases of seven common diseases and 3,000 shared controls Nature 2007 447 7145 661 678 10.1038/nature05911 17554300 PMC2719288

[B19] Festen EA Goyette P Scott R Annese V Zhernakova A Lian J Lefebvre C Brant SR Cho JH Silverberg MS Genetic variants in the region harbouring IL2/IL21 associated with ulcerative colitis Gut 2009 58 6 799 804 10.1136/gut.2008.166918 19201773 PMC2757103

[B20] Liu Y Helms C Liao W Zaba LC Duan S Gardner J Wise C Miner A Malloy MJ Pullinger CR A genome-wide association study of psoriasis and psoriatic arthritis identifies new disease loci PLoS Genet 2008 4 3 e1000041 10.1371/journal.pgen.1000041 18369459 PMC2274885

[B21] van Heel DA Franke L Hunt KA Gwilliam R Zhernakova A Inouye M Wapenaar MC Barnardo MC Bethel G Holmes GK A genome-wide association study for celiac disease identifies risk variants in the region harboring IL2 and IL21 Nat Genet 2007 39 7 827 829 10.1038/ng2058 17558408 PMC2274985

[B22] Zhernakova A Alizadeh BZ Bevova M van Leeuwen MA Coenen MJ Franke B Franke L Posthumus MD van Heel DA Steege G van der Novel association in chromosome 4q27 region with rheumatoid arthritis and confirmation of type 1 diabetes point to a general risk locus for autoimmune diseases Am J Hum Genet 2007 81 6 1284 1288 10.1086/522037 17999365 PMC2276343

[B23] Fedetz M Ndagire D Fernandez O Leyva L Guerrero M Arnal C Lucas M Izquierdo G Delgado C Alcina A Multiple sclerosis association study with the TENR-IL2-IL21 region in a Spanish population Tissue Antigens 2009 74 3 244 247 10.1111/j.1399-0039.2009.01298.x 19523143

[B24] Marquez A Orozco G Martinez A Palomino-Morales R Fernandez-Arquero M Mendoza JL Taxonera C Diaz-Rubio M Gomez-Garcia M Nieto A Novel Association of the Interleukin 2-Interleukin 21 Region With Inflammatory Bowel Disease Am J Gastroenterol 2009 19471255 10.1038/ajg.2009.224

[B25] Adamovic S Amundsen SS Lie BA Gudjonsdottir AH Ascher H Ek J van Heel DA Nilsson S Sollid LM Torinsson Naluai A Association study of IL2/IL21 and FcgRIIa: significant association with the IL2/IL21 region in Scandinavian coeliac disease families Genes Immun 2008 9 4 364 367 10.1038/gene.2008.27 18418394

[B26] Cooper JD Smyth DJ Smiles AM Plagnol V Walker NM Allen JE Downes K Barrett JC Healy BC Mychaleckyj JC Meta-analysis of genome-wide association study data identifies additional type 1 diabetes risk loci Nat Genet 2008 40 12 1399 1401 10.1038/ng.249 18978792 PMC2635556

[B27] Glas J Stallhofer J Ripke S Wetzke M Pfennig S Klein W Epplen JT Griga T Schiemann U Lacher M Novel Genetic Risk Markers for Ulcerative Colitis in the IL2/IL21 Region Are in Epistasis With IL23R and Suggest a Common Genetic Background for Ulcerative Colitis and Celiac Disease Am J Gastroenterol 2009 10.1038/ajg.2009.163 19455118

[B28] Romanos J Barisani D Trynka G Zhernakova A Bardella MT Wijmenga C Six new coeliac disease loci replicated in an Italian population confirm association with coeliac disease J Med Genet 2009 46 1 60 63 10.1136/jmg.2008.061457 18805825

[B29] Garner CP Murray JA Ding YC Tien Z van Heel DA Neuhausen SL Replication of Celiac Disease UK Genome-Wide Association Study Results in a US Population Hum Mol Genet 2009 19648293 10.1093/hmg/ddp364 PMC2758145

[B30] Hemminki K Li X Sundquist J Sundquist K Cancer risks in ulcerative colitis patients Int J Cancer 2008 123 6 1417 1421 10.1002/ijc.23666 18561319

[B31] Hemminki K Li X Sundquist K Sundquist J Cancer risk in hospitalized rheumatoid arthritis patients Rheumatology (Oxford) 2008 47 5 698 701 10.1093/rheumatology/ken130 18378514

[B32] Hemminki K Li X Sundquist J Sundquist K Cancer risks in Crohn disease patients Ann Oncol 2009 20 3 574 580 10.1093/annonc/mdn595 18765463

[B33] Ji J Shu X Li X Sundquist K Sundquist J Hemminki K Cancer risk in hospitalised asthma patients Br J Cancer 2009 100 5 829 833 10.1038/sj.bjc.6604890 19174822 PMC2653753

[B34] Severi G Giles GG Southey MC Tesoriero A Tilley W Neufing P Morris H English DR McCredie MR Boyle P ELAC2/HPC2 polymorphisms, prostate-specific antigen levels, and prostate cancer J Natl Cancer Inst 2003 95 11 818 824 12783937 10.1093/jnci/95.11.818

[B35] Matesanz F Fedetz M Leyva L Delgado C Fernandez O Alcina A Effects of the multiple sclerosis associated -330 promoter polymorphism in IL2 allelic expression J Neuroimmunol 2004 148 1-2 212 217 10.1016/j.jneuroim.2003.12.001 14975604

[B36] Hoffmann SC Stanley EM Darrin Cox E Craighead N DiMercurio BS Koziol DE Harlan DM Kirk AD Blair PJ Association of cytokine polymorphic inheritance and in vitro cytokine production in anti-CD3/CD28-stimulated peripheral blood lymphocytes Transplantation 2001 72 8 1444 1450 10.1097/00007890-200110270-00019 11685118

[B37] Tindall EA Speight G Petersen DC Padilla EJ Hayes VM Novel Plexor SNP genotyping technology: comparisons with TaqMan and homogenous MassEXTEND MALDI-TOF mass spectrometry Hum Mutat 2007 28 9 922 927 10.1002/humu.20533 17458878

[B38] Sham PC Statistics in human genetics. Applications of statistics 1998 London: Arnold

[B39] John S Turner D Donn R Sinnott P Worthington J Ollier WE Hutchinson IV Hajeer AH Two novel biallelic polymorphisms in the IL-2 gene Eur J Immunogenet 1998 25 6 419 420 10.1046/j.1365-2370.1998.00139.x 9949947

[B40] Sawalha AH Kaufman KM Kelly JA Adler AJ Aberle T Kilpatrick J Wakeland EK Li QZ Wandstrat AE Karp DR Genetic association of interleukin-21 polymorphisms with systemic lupus erythematosus Ann Rheum Dis 2008 67 4 458 461 10.1136/ard.2007.075424 17720724

[B41] Asano K Ikegami H Fujisawa T Nishino M Nojima K Kawabata Y Noso S Hiromine Y Fukai A Ogihara T Molecular scanning of interleukin-21 gene and genetic susceptibility to type 1 diabetes Hum Immunol 2007 68 5 384 391 10.1016/j.humimm.2007.01.009 17462506

[B42] Chatterjee R Batra J Ghosh B A common exonic variant of interleukin21 confers susceptibility to atopic asthma Int Arch Allergy Immunol 2009 148 2 137 146 10.1159/000155744 18802358

