# Supplementary material for: The 4q27 locus and prostate cancer risk
Source: BMC Cancer. 2010 Feb 25;10:69. doi: 10.1186/1471-2407-10-69 (PMC2841665; doi:10.1186/1471-2407-10-69)
Supplement: Additional file 1 — Table S1. Characteristics of participants in the Australian Risk Factors for Prostate Cancer Study [file 1471-2407-10-69-S1.DOC]

**Table 1 - Characteristics of participants in the Australian Risk Factors for Prostate Cancer Study**

| **Factor** | **Cases (%)**  **N = 818** | **Controls (%)**  N = 734 |
| --- | --- | --- |
|  |  |  |
| Reference Age |  |  |
| < 55 yrs | 511 (62) | 485 (66) |
| 55 – 59 yrs | 111 (14) | 131 (18) |
| 60 – 69 yrs | 196 (24) | 118 (16) |
|  |  |  |
| Country of birth |  |  |
| Australia | 570 (70) | 479 (65) |
| Overseas | 247 (30) | 255 (35) |
|  |  |  |
| Number of affected first-degree relatives |  |  |
| None | 673 (82) | 694 (95) |
| One | 124 (15) | 39 (5) |
| Two or more | 21 (3) | 1 (0) |
|  |  |  |
| Tumour Stage |  |  |
| I-II | 561 (69) | – |
| III-IV | 253 (31) | – |
|  |  |  |
| Tumour Grade |  |  |
| Moderate (Gleason score 5-7) | 597 (73) | – |
| High (Gleason score 8-10) | 221 (27) | – |
|  |  |  |
|  |  |  |
