# Supplementary material for: The 4q27 locus and prostate cancer risk
Source: BMC Cancer. 2010 Feb 25;10:69. doi: 10.1186/1471-2407-10-69 (PMC2841665; doi:10.1186/1471-2407-10-69)
Supplement: Additional file 2 — Table S2. Allele and genotype distributions for seven variants across chromosome 4q27 and prostate cancer risk [file 1471-2407-10-69-S2.DOC]

## Table 2 - Allele and genotype distributions for seven variants across chromosomal region 4q27 and prostate cancer risk

| Variant ID | Nucleotide substitution | gene | Minor allele frequencies (N) | | Per allele  OR (95% CI) | P | Heterozygous OR (95% CI) | Homozygous OR (95% CI) | P-value  (recessive) | P-value  (dominant) |
| --- | --- | --- | --- | --- | --- | --- | --- | --- | --- | --- |
|  |  |  | Cases | Controls |  |  |  |  |  |  |
| rs2069762 | T>G | *IL-2* (promoter) | 0.29 (818) | 0.31 (734) | 0.92 (0.79-1.07) | 0.3 | 0.93 (0.75-1.15) | 0.82 (0.57-1.17) | 0.3 | 0.4 |
| rs13151961 | A>G | *KIAA1109* | 0.16 (789) | 0.16 (722) | 0.97 (0.80-1.18) | 0.8 | 1.04 (0.83-1.31) | 0.70 (0.36-1.35) | 0.3 | >0.9 |
| rs13119723 | A>G | *KIAA1109* | 0.16 (790) | 0.17 (718) | 0.95 (0.79-1.16) | 0.6 | 1.03 (0.82-1.29) | 0.68 (0.36-1.27) | 0.2 | >0.9 |
| rs17388568 | G>A | *TENR* | 0.27 (792) | 0.26 (721) | 1.08 (0.92-1.27) | 0.3 | 1.12 (0.90-1.38) | 1.11 (0.74-1.66) | 0.8 | 0.3 |
| rs3136534 | A>C | *IL-2* (3’) | 0.34 (792) | 0.31 (725) | 1.16 (1.00-1.35) | 0.06 | 1.22 (0.98-1.51) | 1.28 (0.91-1.80) | 0.4 | 0.05 |
| rs6822844 | G>T | *IL-21* (3’) | 0.16 (793) | 0.16 (725) | 1.00 (0.83-1.22) | >0.9 | 1.05 (0.84-1.32) | 0.84 (0.45-1.55) | 0.5 | 0.8 |
| rs6840978 | C>T | *IL-21* (5’) | 0.20 (793) | 0.20 (724) | 0.96 (0.80-1.14) | 0.6 | 0.97 (0.78-1.21) | 0.90 (0.55-1.47) | 0.7 | 0.7 |
