# Supplementary material for: The 4q27 locus and prostate cancer risk
Source: BMC Cancer. 2010 Feb 25;10:69. doi: 10.1186/1471-2407-10-69 (PMC2841665; doi:10.1186/1471-2407-10-69)
Supplement: Additional file 3 — Table S3. Association between variants and prostate cancer risk by family history of prostate cancer [file 1471-2407-10-69-S3.DOC]

**Table 3** - **Association between chromosome 4q27 variants and prostate cancer risk by family history of prostate cancer**

|  |  | No affected relatives | | 1+ affected relative | |  |
| --- | --- | --- | --- | --- | --- | --- |
|  | genotype | N cases (%)/  N controls (%) | Per allele  OR (95% CI)^1^ | N cases (%)/  N controls (%) | Per allele  OR (95% CI) ^1^ | P-interaction |
| rs2069762 | TT  TG  GG | 334 (50)/ 335 (48)  278 (41)/ 288 (42)  61 ( 9)/ 71 (10) | 0.94 (0.80-1.11) | 77 (53)/ 17 (43)  56 (39)/ 18 (45)  12 ( 8)/ 5 (13) | 0.71 (0.42-1.20) | 0.3 |
| rs13151961 | AA  AG  GG | 466 (71)/ 477 (70)  174 (27)/ 187 (27)  13 ( 2)/ 20 ( 3) | 0.91 (0.74-1.12) | 87 (64)/ 30 (79)  46 (34)/ 7 (18)  3 ( 2)/ 1 ( 3) | 1.82 (0.83-3.96) | 0.08 |
| rs13119723 | AA  AG  GG | 467 (71)/ 470 (69)  173 (26)/ 187 (28)  15 ( 2)/ 23 ( 3) | 0.89 (0.72-1.09) | 87 (64)/ 33 (84)  45 (33)/ 5 (13)  3 ( 2)/ 1 ( 3) | 2.37 (1.01-5.57) | 0.02 |
| rs17388568 | GG  GA  AA | 348 (53)/ 380 (56)  257 (39)/ 256 (38)  51 ( 8)/ 46 ( 7) | 1.10 (0.93-1.30) | 69 (51)/ 19 (49)  60 (44)/ 16 (41)  7 ( 5)/ 4 (10) | 0.83 (0.47-1.47) | 0.4 |
| rs3136534 | AA  AC  CC | 288 (44)/ 334 (49)  290 (44)/ 284 (41)  77 (12)/ 68 (10) | 1.16 (0.99-1.36) | 55 (40)/ 17 (44)  69 (50)/ 18 (46)  13 ( 9)/ 4 (10) | 1.07 (0.61-1.87) | 0.8 |
| rs6822844 | GG  GT  TT | 463 (71)/ 478 (70)  176 (27)/ 187 (27)  16 ( 2)/ 21 ( 3) | 0.94 (0.77-1.16) | 89 (64)/ 31 (79)  45 (33)/ 7 (18)  4 ( 3)/ 1 ( 3) | 1.85 (0.86-3.97) | 0.08 |
| rs6840978 | CC  CT  TT | 434 (66)/ 437 (64)  195 (30)/ 215 (31)  27 ( 4)/ 33 ( 5) | 0.91 (0.76-1.10) | 83 (61)/ 28 (72)  47 (34)/ 10 (26)  7 ( 5)/ 1 ( 3) | 1.57 (0.80-3.09) | 0.1 |

^1^Estimates obtained from a logistic model that includes the interaction between genotype and family history of prostate cancer. The log odds ratio for the group with a family history was obtained by adding the coefficient for the genotype and the coefficient for the interaction between genotype and family history
